# Supplementary material for: Transcriptional landscapes underlying Notch-induced lineage conversion and plasticity of mammary basal cells
Source: EMBO J. 2025 Apr 4;44(10):2827–55. doi: 10.1038/s44318-025-00424-1 (PMC12084385; doi:10.1038/s44318-025-00424-1)
Supplement: Supplementary file 1 — Appendix [file 44318_2025_424_MOESM1_ESM.pdf]

Appendix

Table of content:

Appendix Table S1: Number of cells index-sorted per time and 96-well plate p. 2
Appendix Table S2: List of genes used for cell cycle score p. 3
Appendix Table S3: List of genes correlated with pseudotime analysis p. 4

Appendix Table S1: Number of cells index-sorted per time and 96-well plate

	Chase	LC GFP+	BC GFP+	Inter GFP+	LC GFP-	BC GFP-
**SMACre/N1ICD**	1w	6	73	11	6	
**SMACre/N1ICD**	3w	34	12	44	6	
**SMACre/N1ICD**	3w	30	34	26	6	
**SMACre/N1ICD**	3w	0	0	60	6	6
**K5Cre/N1ICD**	3w	6	6	66	6	6
**K5Cre/N1ICD**	3w	6	6	66	6	6
**SMACre/N1ICD**	4w	22	17	51	6	
**SMACre/N1ICD**	6w	84			12	

Appendix Table S2: List of genes used for the cell cycle score

Adam17	Cdc14b	Dbf4	Hyal1	Med1	Phb2	Rock2	Thoc1
Adamts1	Cdc16	Ddr2	Igf1	Mepce	Phip	Rpl17	Thoc5
Akt1	Cdc20	Ddx3x	Igf1r	Met	Piwil2	Rptor	Tmod3
Anapc5	Cdc23	Ddx39b	Igf2	Msx1	Pkn2	Rrm1	Tnf
Anapc7	Cdc25a	Dtl	Incenp	Msx2	Pkp4	Rrm2	Tpr
Anapc11	Cdc25b	Dync1h1	Ino80	Mta3	Plcb1	Rrm2b	Trp63
Ankrd17	Cdc25c	Dync1li1	Insr	Mtbp	Plcg2	Sass6	Ube2b
Anp32b	Cdc42	Dyrk3	Kat2b	Ncapd2	Plk4	Sfpq	Ube2c
Anxa1	Cdc73	E2f7	Kat5	Ncapd3	Plrg1	Sgo2a	Ube2e2
Apex1	Cdca5	E2f8	Kcnn4	Ncapg	Poc1a	Sh2b1	Ubxn2b
App	Cdca8	Ect2	Kif3b	Ncapg2	Poldip2	Sin3a	Usp19
Atad5	Cdk1	Edn1	Kif14	Ncaph	Ppp1r10	Sirt2	Vps4b
Atrx	Cdk4	Egf	Kif20b	Ncaph2	Ppp1r35	Slf1	Wiz
Aurka	Cenpe	Egfr	Kif23	Ndc80	Prdm9	Slf2	Wnk1
Aurkb	Cenpj	Eif4g1	Klhl18	Npm1	Prkce	Smarcd3	Wnt4
Azin1	Cenpv	Eif4g3	Kmt2e	Npm2	Rab11a	Smc2	Wnt5a
Becn1	Cep120	Epgn	Knl1	Npr2	Rab11fip4	Smc4	Xrcc3
Birc5	Cep295	Ereg	Larp7	Nsfl1c	Racgap1	Smc5	
Brd4	Chek2	Ezh2	Lef1	Nsmce2	Rad18	Smc6	
Btc	Chmp3	Fam83d	Lmnb1	Nudt16	Rad21	Smpd3	
Bub1	Cit	Fbxo5	Lsm10	Numa1	Rad51ap1	Sox15	
Camk2b	Cpsf3	Fen1	Lsm11	Nup62	Rad51b	Spag5	
Camk2d	Crebbp	Fgfr1	Macroh2a1	Nusap1	Rad51c	Spast	
Ccn2	Csf1r	Gen1	Mad1l1	Orc1	Ranbp1	Sphk1	
Ccnb1	Cspp1	Gipc1	Mad2l1	Paf1	Rb1	Stil	
Ccnd1	Cul3	Gja1	Mad2l1bp	Pbx1	Rcc2	Stxbp4	
Ccnd2	Cul4a	Gli1	Map3k20	Pcid2	Rdx	Tbx2	
Cdc6	Cul4b	Gpsm2	Map10	Pdgfb	Rgcc	Tert	
Cdc7	Cxcr5	Hnrnpu	Mapk15	Pdgfrb	Rhno1	Tgfa	
Cdc14a	D1Pas1	Hspa2	Mdm2	Pebp1	Rhoa	Tgfb1	

Appendix Table S3: LIst of genes correlated with pseudotime analysis

Bas_early		Bas_late	Int_early	Int_late	Lum_early		Lum_late
Myh11	Cd109	Adgra3	Krt16	Krt79	Aldh1a3	Rftn1	Plin2
Slpi	Gpr3	Dennd2c	Crispld2	Alox12e	Car2	Nectin4	Csn3
Postn	Gfra2	Gpc1	Gltp	Dsc2	Scd1	Atp6v1b1	Lrg1
Hmox1	Mical2	Tppp3	Cyp1b1	Pdk4	Cd14	Plekha6	Slc28a3
Acta2	Grik3	Cdk18	Ptn	Aldh3a2	Prlr	Slc5a8	Trf
Ptx3	Cxcl14	Lrp4	Cstb	Cnmd	Ltf	Tnf	Btn1a1
Col4a2	Angptl2	Pdpn	St3gal1	Epha4	Cpe	Hid1	Igfbp5
Flnc	Gucy1a1	Zfp703	A130010J15R	Acsl5	Txnip	Rhpn2	C3
Adamts1	Prickle2	Itga3	Sdc1	Gabrp	Cldn1	Pard6b	Errfi1
Dll1	Jag1	Ndrg2	Psat1	Plk2	Ceacam1	Mast3	Srxn1
Col6a1	Matn2	Sparc	Ptpn13	Zfp750	Apod	Cldn7	Dusp5
Eln	Ltbp4	Ston2	Pkp1	Mcm3	Ermp1	Thsd4	Cck
Socs3	Aebp1	Chadl	Tubb5	H1f4	Clu	Cldn3	Clic6
Cnn1	Perp	Nfil3	Zcchc24	Mcm2	Nupr1	Pkp2	Muc1
Mmp2	Ecrg4	Krt17	Nebl	Trim16	Slc5a1	Cd55	Tubb2a
Gem	Lifr	Soga1	Sipa1l1	Palmd	Aqp5	Ntn1	Xdh
Col6a2	Thbs1	Bmp7	S100a14	Ece1	Lgals3	Tspan33	Pim1
Gas1	Tns1	Creb3l2	Bach1	Blnk	Dbi	Itpr2	Lcn2
Nrg1	Rnf24	Smim3	Bahc	Acot1	Bglap3	Tacstd2	Lipa
Myl9	Rnd3	Phlda3		Hspa4l	Fcgbp	Mal2	Muc20
Col14a1	Scube3	Obsl1		Osbpl3	Ckmt1	Jun	
Wnt11	Panx1	Trim29		Csmd1	Dmkn	Ccrl2	
Oxtr	Adamts2	Fgfr2		Ccnd1	Cytip	Dock8	
Slc43a3	Frmd4a	P3h2		Myo5b	Tmprss2	Dhx32	
Mcam	Nckap5l	Dusp7		S100a6	Piezo1	Flnb	
Col12a1	Dsp	Syne1		Tmsb4x	Gm43305	Rab11fip1	
Peg3	Cnp	Itga9		Scara3	Krt19	Ppfibp2	
Vegfa	Palld	Slc25a5			Wnt7b	Krt18	
Serpinf1	Pla2g7	Slc39a10			Tfcp2l1	Trpm6	
Timp3	Mylk	Pard6g			Qsox1	Basp1	
Cntn2	Pcsk5	Tle1			Kcnn4	Elf5	
Sgk1	Arl4c				Anxa1	Ptk2b	
Hmcn1	Lrp1				Tnfaip2	Itih5	
Pmp22	Ptpre				Itgb6	Atp10b	
Scn7a	Msrb3				Spint1	Scara5	
Vim	Synpo				Vill		
Cxcl12	Jag2				Tuft1		
Ackr3	Lamb1				Ly6d		
Fbn1	Clip3				Cp		
Lama3	Axl				Cldn4		
Itga5	Lamb3				Mgst1		
Lgr5	Fstl1				Mboat1		
Bgn	Pros1				Sectm1b		
Hspg2	Col17a1				Krt8		
Ctnnal1	Sfrp1				Grhl1		
Snai2	Man1a				Arhgef6		
Col18a1	Apobec1				Spns2		
Col16a1	Sphk1				Wfdc18		
Icam1	Cav1				Elf3		
Tgfa	Kif26b				Pip5k1c		
Tpbg	Tril				Gprc5a		
	Dpysl3						
	Antxr1						
	Prnp						
	Dkk3						
	Sdk2						
	Smad7						
	Map1b						
	Bmp1						
	Fat2						
	Ankrd44						
	Tsc22d1						
	Sema5a						
	Fblim1						
	Ptpn14						
	Cpxm2						
	Slc20a2						
	Dst						
	Sorbs1						
	Kirrel						
	Dclk1						
