# Supplementary material for: Transcriptional landscapes underlying Notch-induced lineage conversion and plasticity of mammary basal cells
Source: EMBO J. 2025 Apr 4;44(10):2827–55. doi: 10.1038/s44318-025-00424-1 (PMC12084385; doi:10.1038/s44318-025-00424-1)
Supplement: Supplementary file 3 — Expanded View Figures [file 44318_2025_424_MOESM3_ESM.pdf]

Expanded View Figures

Figure EV1. Transcriptional signatures characterizing the different cell clusters identified by UMAP analysis.

Related to Fig. 2. (**A**) Representative images of SMACre^{ERT2}/mTmG mammary gland (MG), lacrimal gland (LG), salivary gland (SG), and K5Cre^{ERT2}/mTmG prostate sections induced at P21 and analyzed 6 weeks later by immunofluorescence for mGFP (yellow) and the basal marker α-SMA (white), demonstrating that α-SMA^{pos} cells are exclusively BCs, indicating unipotency. Scale bars represent 50 µm. (**B**) Gating strategy used for cell sorting experiments. Doublets, Lin^{pos} and dead cells (DAPI^{pos}) were excluded from further analysis. (**C**) FACS plots showing the gates defining luminal (EpCAM^{high}/Cd49f^{low}), intermediate (EpCAM^{med}/Cd49f^{med}), and basal (EpCAM^{low}/Cd49f^{high}) sorted cells from SMACre^{ERT2}/N1ICD mammary glands induced at P21 and chased for 1, 3, or 6 weeks, that were used for SMARTseqV2. The percentages of each population are indicated for one representative experiment. (**D**) UMAP plot showing the distribution of each sequenced cell in the different clusters based on the Cre mouse used to target them (K5Cre^{ERT2} or SMACre^{ERT2}). (**E**) UMAP plots show the expression of well-defined markers for basal (*Krt5, Krt14*), luminal (*Krt8, Krt19*), and HR^{pos} (*Esr1, Pgr*) cells. (**F**) Violin plots representing basal and luminal scores, based on signatures from (Kendrick et al, 2008) for each cluster. (**G**) UMAP plot indicating the cell cycle phases for each sequenced cell. The boxed cells correspond to the proliferative population highlighted in Fig. 2E. (**H**) UMAP plots are color-coded according to the expression of the single-cell G2M and S score. (**I**) Heatmap showing the genes presenting a high expression in the proliferative group. (**J**) Proportion of cells in different cell cycle phases (G1, G2M, or S) in each cluster.

© The Author(s)

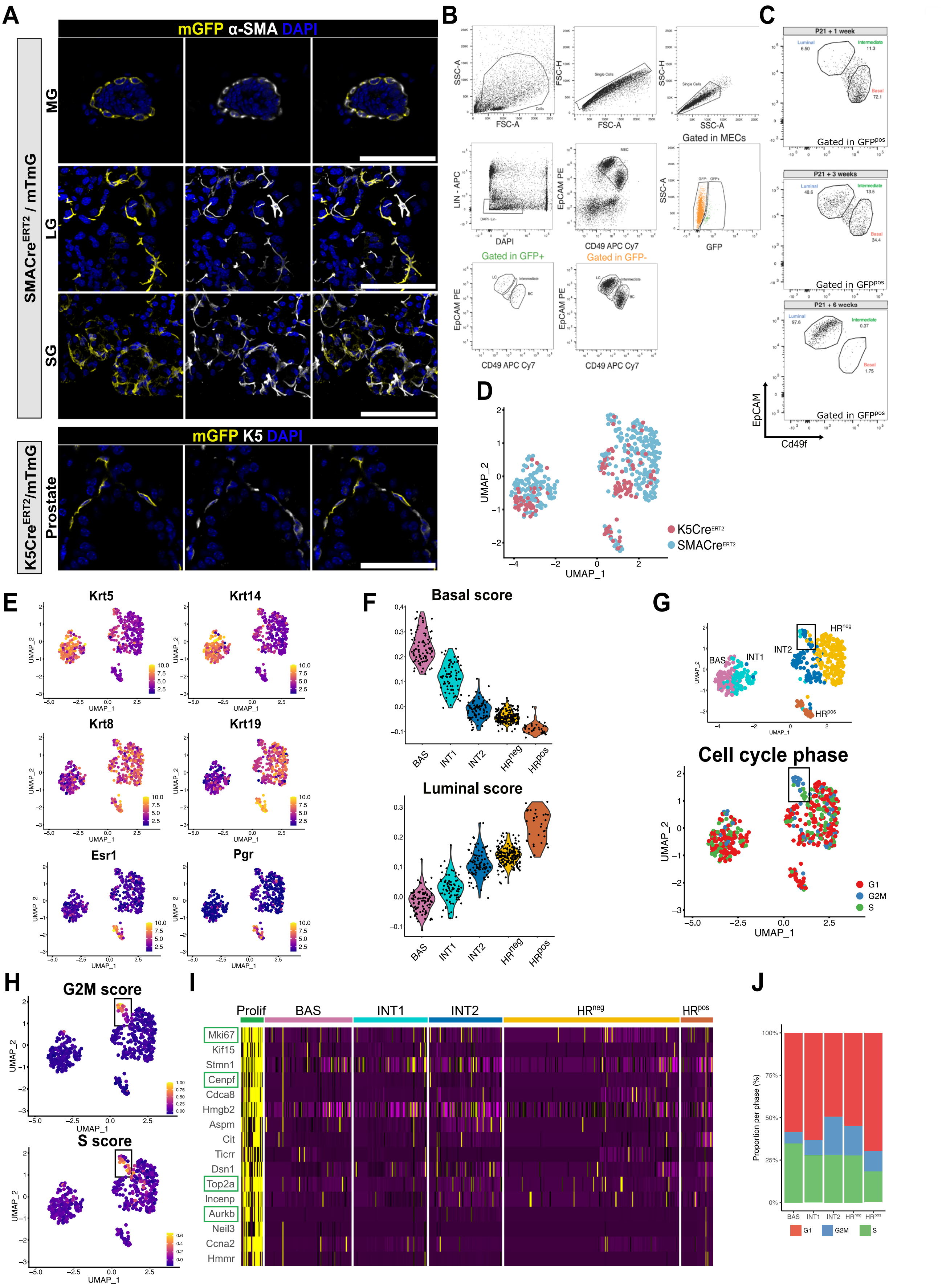
A
mGFP α-SMA DAPI
SMACreERT2 / mTmG
MG
LG
SG
mGFP K5 DAPI
K5CreERT2/mTmG
Prostate
B
SSC-A
FSC-A
FSC-H
SSC-H
Gated in MECs
LIN - APC
DAPI
EpCAM PE
CD49 APC Cy7
GFP
Gated in GFP+
Gated in GFP-
C
P21 + 1 week
P21 + 3 weeks
P21 + 6 weeks
Gated in GFPpos
EpCAM
Cd49f
D
UMAP_2
UMAP_1
K5CreERT2
SMACreERT2
E
Krt5
Krt14
Krt8
Krt19
Esr1
Pgr
F
Basal score
Luminal score
BAS
INT1
INT2
HRneg
HRpos
G
Cell cycle phase
G1
G2M
S
H
G2M score
S score
I
Prolif
Mki67
Kif15
Stmn1
Cenpf
Cdca8
Hmgb2
Aspm
Cit
Ticrr
Dsn1
Top2a
Incenp
Aurkb
Neil3
Ccna2
Hmmr
J
Proportion per phase (%)

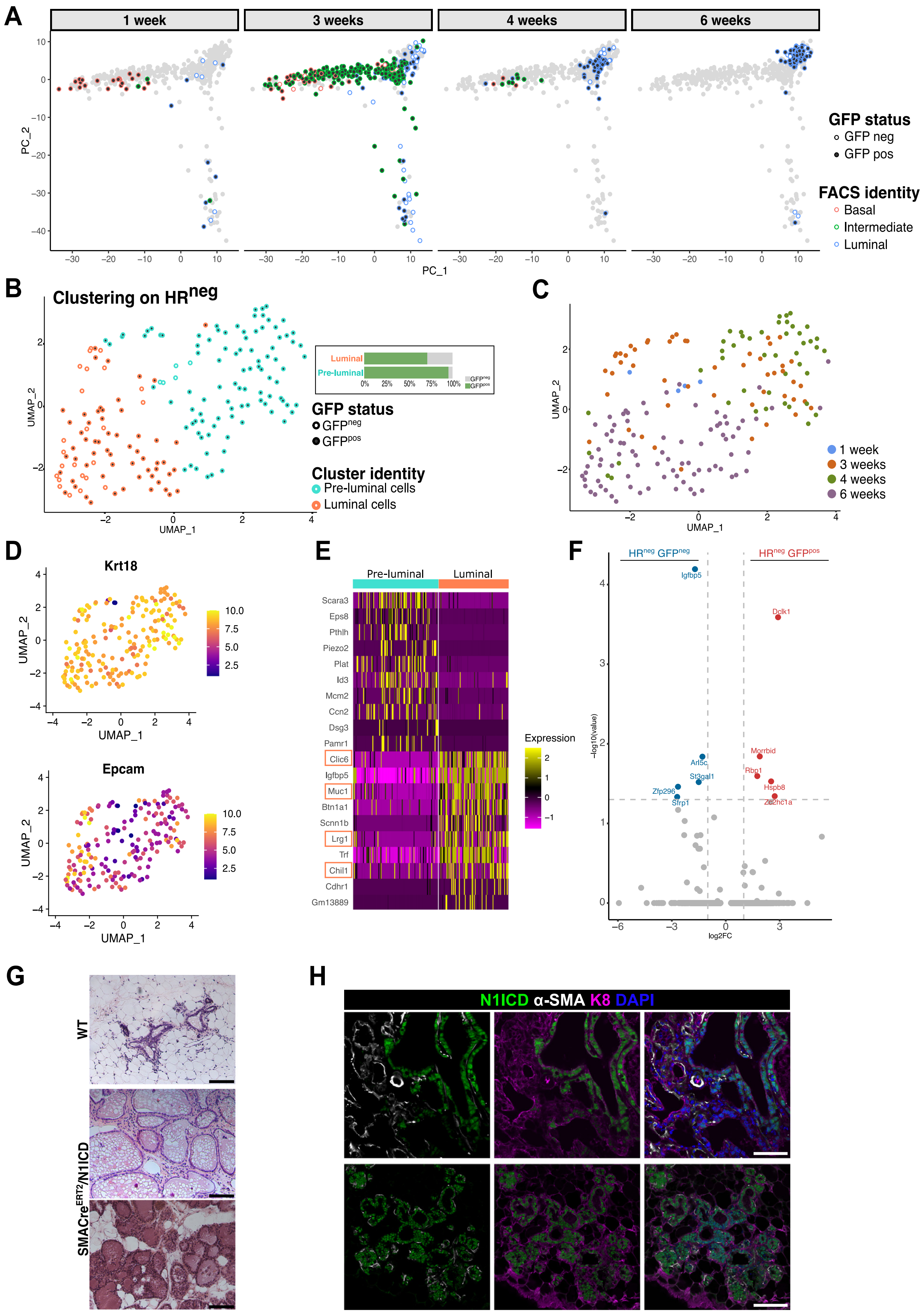
A
1 week
3 weeks
4 weeks
6 weeks
PC_2
PC_1
GFP status
GFP neg
GFP pos
FACS identity
Basal
Intermediate
Luminal
B
Clustering on HRneg
UMAP_2
UMAP_1
Luminal
Pre-luminal
GFP status
GFPneg
GFPpos
Cluster identity
Pre-luminal cells
Luminal cells
C
1 week
3 weeks
4 weeks
6 weeks
D
Krt18
Epcam
E
Pre-luminal
Luminal
Scara3
Eps8
Pthlh
Piezo2
Plat
Id3
Mcm2
Ccn2
Dsg3
Pamr1
Clic6
Igfbp5
Muc1
Btn1a1
Scnn1b
Lrg1
Trf
Chil1
Cdhr1
Gm13889
Expression
F
HRneg GFPneg
HRneg GFPpos
Igfbp5
Dclk1
Morrbid
Arl5c
Rbp1
St3gal1
Hspb8
Zfp296
Zc2hc1a
Sfrp1
-log10(value)
log2FC
G
WT
SMACreERT2/N1CD
H
N1ICD α-SMA K8 DAPI

 © The Author(s)

Figure EV2. Lineage trajectories of cell fate switch and associated transcriptional features.

Related to Fig. 3. (**A**) PCA plots highlighting changes in cell transcriptional state along the basal-luminal differentiation trajectory, at different timepoints following N1ICD activation. Colored dots indicate the FACS gate information for each index-sorted cell, based on the cell surface markers EpCAM and Cd49f. (**B**) UMAP refined analysis of the HR^{neg} cluster indicating GFP^{pos} and GFP^{neg} cells. The two new resulting subclusters appearing are indicated by different colors (turquoise and orange). The boxed graph represents the percentage of GFP^{pos} (green) and GFP^{neg} (gray) cells for each cluster, showing the predominance of GFP^{pos} mutant cells in the pre-luminal cluster. (**C**) UMAP representation of the distribution of mutant $nGFP^{pos}$ cells belonging to the HR^{neg} cluster color-coded based on the different timepoints after N1ICD induction, as indicated. (**D**) UMAP representations of the two luminal subclusters show the expression levels of two luminal-specific genes (*Krt18* and *Epcam*) for each cell. (**E**) Heatmap showing differentially expressed genes (DEGs) distinguishing the pre-luminal and luminal clusters. (**F**) Volcano Plot of DEGs between HR^{neg} GFP^{pos} (170 cells) (red dots) and HR^{neg} GFP^{neg} (34 cells) (blue dots) cells. (**G**) Hematoxylin and Eosin staining of WT (wild-type) mammary gland and mammary tumor sections following N1ICD activation. Wild-type mouse mammary glands were collected after one pregnancy, while tumors were harvested after three pregnancies, followed by 10 to 15 days of involution. (**H**) Immunofluorescence anti-GFP (corresponding to N1ICD), α-SMA, and K8 on sections of mammary tumors developed upon N1ICD activation, three pregnancies, and 10 days of involution showing the clonal expansion of mutant $nGFP^{pos}$ cells. Nuclei are stained with DAPI. Scale bars represent 100 µm in (**G**, **H**).

© The Author(s)

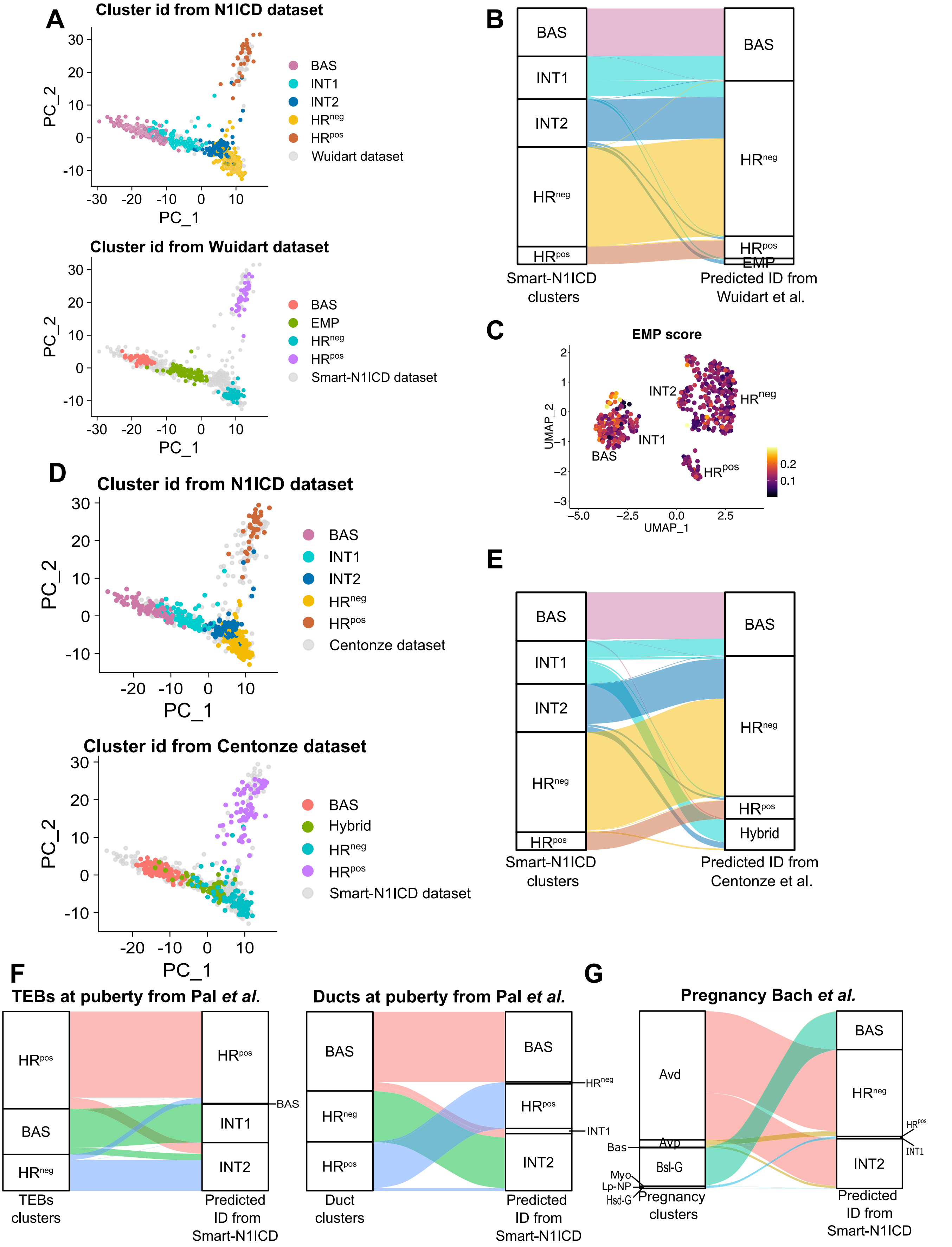
A
Cluster id from N1ICD dataset
BAS
INT1
INT2
HRneg
HRpos
Wuidart dataset
Cluster id from Wuidart dataset
BAS
EMP
HRneg
HRpos
Smart-N1ICD dataset
PC_1
PC_2
B
Smart-N1ICD clusters
Predicted ID from Wuidart et al.
C
EMP score
UMAP_1
UMAP_2
D
Cluster id from N1ICD dataset
Centonze dataset
Cluster id from Centonze dataset
Hybrid
E
Predicted ID from Centonze et al.
F
TEBs at puberty from Pal et al.
Ducts at puberty from Pal et al.
TEBs clusters
Duct clusters
Predicted ID from Smart-N1ICD
G
Pregnancy Bach et al.
Avd
Avp
Bsl-G
Bas
Myo
Lp-NP
Hsd-G
Pregnancy clusters

 © The Author(s)

Figure EV3. Integration of SMART-N1ICD dataset with scRNAseq from embryonic and adult hybrid mammary cells.

Related to Fig. 3. (**A**) PCA plots showing the integration of our data (SMART-N1ICD) and the dataset from Wuidart et al (Wuidart et al, 2018). (**B**) Alluvium plots showing label transfer of each cell cluster using the Wuidart dataset as reference. (**C**) UMAP plot representing enrichment for the EMP score derived from Wuidart et al, dataset. Purple represents the lowest EMP score and yellow the highest EMPs score. (**D**) PCA plots showing the integration of our data (SMART-N1ICD) and the dataset from Centonze et al, (Centonze et al, 2020). (**E**) Alluvium plots showing label transfer of each cell cluster using the Centonze dataset as reference. (**F**) Alluvium plots showing label transfer of each cell cluster in the Pal et al, dataset either from TEBs (left) or from pubertal ducts (right) using our dataset (Smart-N1ICD) as reference. (**G**) Alluvium plot showing label transfer of each cell cluster in the Bach et al., dataset performed at pregnancy using our dataset (Smart-N1ICD) as reference.

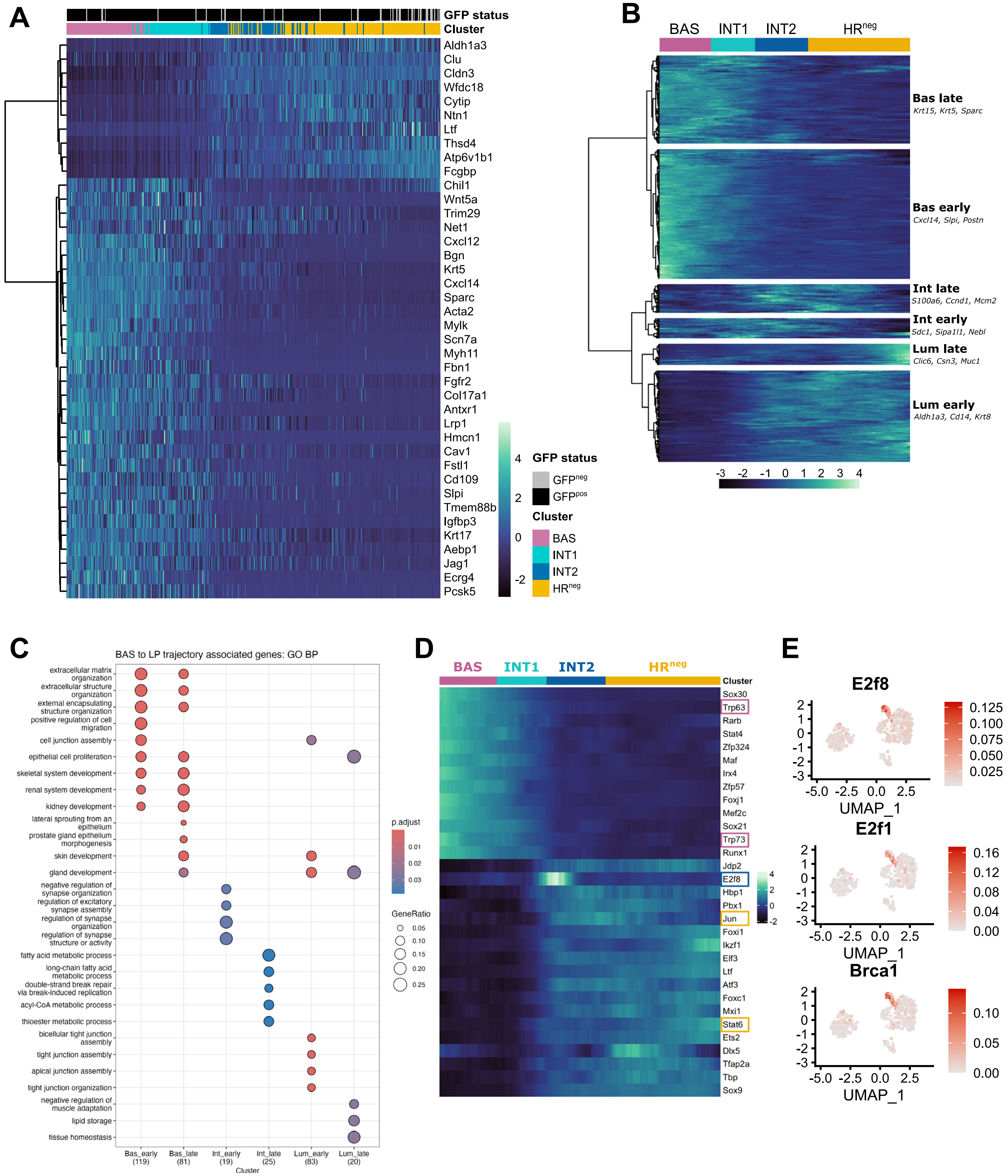
A
GFP status
Cluster
Aldh1a3
Clu
Cldn3
Wfdc18
Cytip
Ntn1
Ltf
Thsd4
Atp6v1b1
Fcgbp
Chil1
Wnt5a
Trim29
Net1
Cxcl12
Bgn
Krt5
Cxcl14
Sparc
Acta2
Mylk
Scn7a
Myh11
Fbn1
Fgfr2
Col17a1
Antxr1
Lrp1
Hmcn1
Cav1
Fstl1
Cd109
Slpi
Tmem88b
Igfbp3
Krt17
Aebp1
Jag1
Ecrg4
Pcsk5
4
2
0
-2
GFP status
GFPneg
GFPpos
Cluster
BAS
INT1
INT2
HRneg
B
BAS
INT1
INT2
HRneg
Bas late
Krt15, Krt5, Sparc
Bas early
Cxcl14, Slpi, Postn
Int late
S100a6, Ccnd1, Mcm2
Int early
Sdc1, Sipa1l1, Nebl
Lum late
Clic6, Csn3, Muc1
Lum early
Aldh1a3, Cd14, Krt8
-3 -2 -1 0 1 2 3 4
C
BAS to LP trajectory associated genes: GO BP
extracellular matrix organization
extracellular structure organization
external encapsulating structure organization
positive regulation of cell migration
cell junction assembly
epithelial cell proliferation
skeletal system development
renal system development
kidney development
lateral sprouting from an epithelium
prostate gland epithelium morphogenesis
skin development
gland development
negative regulation of synapse organization
regulation of excitatory synapse assembly
regulation of synapse organization
regulation of synapse structure or activity
fatty acid metabolic process
long-chain fatty acid metabolic process
double-strand break repair via break-induced replication
acyl-CoA metabolic process
thioester metabolic process
bicellular tight junction assembly
tight junction assembly
apical junction assembly
tight junction organization
negative regulation of muscle adaptation
lipid storage
tissue homeostasis
Bas_early (119)
Bas_late (81)
Int_early (19)
Int_late (25)
Lum_early (83)
Lum_late (20)
Cluster
p.adjust
0.01
0.02
0.03
GeneRatio
0.05
0.10
0.15
0.20
0.25
D
BAS
INT1
INT2
HRneg
Cluster
Sox30
Trp63
Rarb
Stat4
Zfp324
Maf
Irx4
Zfp57
Foxj1
Mef2c
Sox21
Trp73
Runx1
Jdp2
E2f8
Hbp1
Pbx1
Jun
Foxi1
Ikzf1
Elf3
Ltf
Atf3
Foxc1
Mxi1
Stat6
Ets2
Dlx5
Tfap2a
Tbp
Sox9
4
2
0
-2
E
E2f8
0.125
0.100
0.075
0.050
0.025
E2f1
0.16
0.12
0.08
0.04
0.00
Brca1
0.10
0.05
0.00
-5.0 -2.5 0.0 2.5
UMAP_1

 © The Author(s)

Figure EV4. Pseudotime ordering identifies the transcriptional signatures associated with the progressive lineage transition from BCs to LCs.

Related to Fig. 3. (**A**) Heatmap illustrating the top 40 genes exhibiting a differential pattern of expression along the pseudotime from basal to luminal (HR^{neg}) identity. The clusters are color-coded, as in Fig. 2B. (**B**) Heatmap showing the dynamic expression profile of each gene towards the lineage switch trajectory, distinguishing six different patterns of expression along the process of basal to luminal transition. (**C**) GO terms associated with the genes defining the six groups in (**B**). *p* values were defined using one-sided Fisher's exact statistical test. (**D**) Heatmap showing the top transcriptional regulons specific to each cluster (based on RSS analysis), plotted along the pseudotime trajectory 1. Boxed genes are described in the text. (**E**) UMAP plots showing the expression of regulons specific to cluster INT2, based on Fig. 3E.

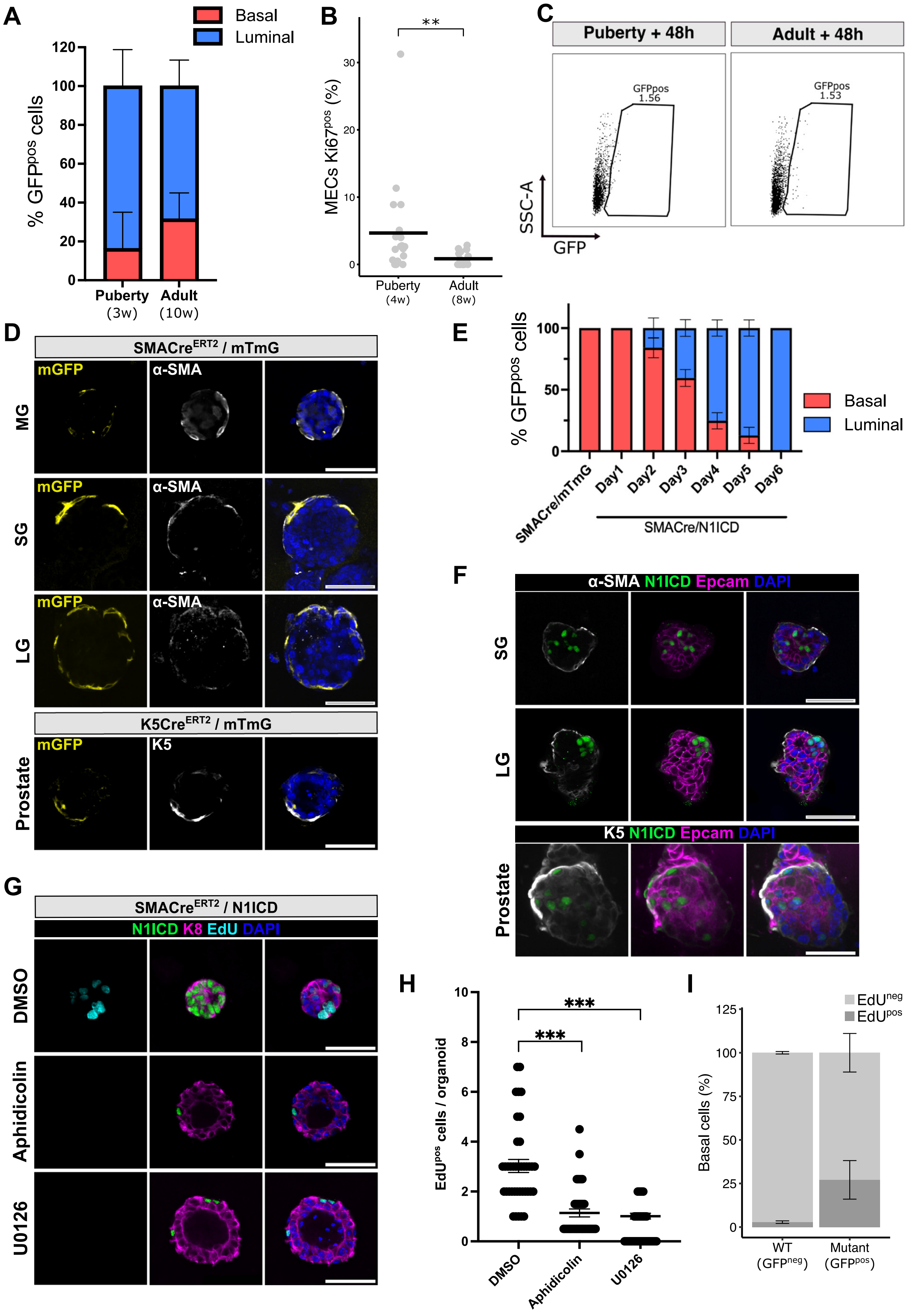
A
Basal
Luminal
% GFPpos cells
Puberty (3w)
Adult (10w)
B
**
MECs Ki67pos (%)
Puberty (4w)
Adult (8w)
C
Puberty + 48h
Adult + 48h
GFPpos 1.56
GFPpos 1.53
SSC-A
GFP
D
SMACreERT2 / mTmG
mGFP
α-SMA
MG
SG
LG
K5CreERT2 / mTmG
K5
Prostate
E
% GFPpos cells
SMACre/mTmG
Day1
Day2
Day3
Day4
Day5
Day6
SMACre/N1ICD
Basal
Luminal
F
α-SMA N1ICD Epcam DAPI
K5 N1ICD Epcam DAPI
SG
LG
Prostate
G
SMACreERT2 / N1ICD
N1ICD K8 EdU DAPI
DMSO
Aphidicolin
U0126
H
***
***
EdUpos cells / organoid
DMSO
Aphidicolin
U0126
I
EdUneg
EdUpos
Basal cells (%)
WT (GFPneg)
Mutant (GFPpos)

 © The Author(s)

Figure EV5. Lineage switch and BCs unipotency are recapitulated in organoids.

Related to Figs. 4, 5. (**A**) Quantification of the proportion of GFP^{pos} cells in basal and luminal FACS gates after induction at pre-puberty (3w) or adulthood (10w), showing the incomplete cell fate switch after a 6-week chase in adult mice. Data were displayed as mean ± SEM, and represent at least three mice. (**B**) Percentage of Ki67-positive mammary epithelial cells (MECs) in pubertal (4w) or adult (8w) mice. $n = 3$ mice with a total of 16 sections for pubertal mice and 19 sections for adult mice. p value = 0.009 defined by t-test. (**C**) FACS plots showing the percentage of mutant GFP^{pos} cells after 48 h induction in pubertal or adult mice, demonstrating invariant recombination efficiency. (**D**) Representative immunofluorescence images showing mGFP, α-SMA, and K8 expression, 6 days after 4-OHT induction of $SMACre^{ERT2}$/mTmG control organoids. Scale bar 50 µm. (**E**) Quantification of the proportion of mutant $nGFP^{pos}$ basal and luminal cells per organoid at the indicated times after induction in $SMACre^{ERT2}$/N1ICD mice or 6 days after tamoxifen in $SMACre^{ERT2}$/mTmG control animals. Error bars represent mean ± SEM. A minimum of 18 organoids were analyzed for each condition and included three independent experiments. (**F**) Representative images of $SMACre^{ERT2}$/N1ICD (SG and LG) or $K5Cre^{ERT2}$/ N1ICD (prostate) organoids showing mutant cells ($nGFP^{pos}$), featuring the expression of the luminal marker Epcam and the absence of basal markers (α-SMA or K5, as indicated). Scale bar 50 µm. (**G**) Representative images of EdU staining in $SMACre^{ERT2}$/N1ICD organoids treated with DMSO, Aphidicolin, and U0126. Scale bar represents 50 µm. (**H**) Quantification of EdU^{pos} cells per organoids after 6 days in culture with DMSO, Aphidicolin, or U0126. *** indicates p value <0.0001 (p value = 2.04e-10 for DMSO/Aphidicolin and 2.04e-10 for DMSO/U0126, using Wilcoxon test). Data were displayed as mean ± SEM and represented three independent experiments with at least 40 organoids in total. (**I**) Quantification of the proportion of EdU positive or negative WT (GFP^{neg}) or mutant (GFP^{pos}) BCs in organoids 3 days after induction. Data were displayed as mean ± SEM and 19 organoids were analyzed from three independent experiments.

© The Author(s)

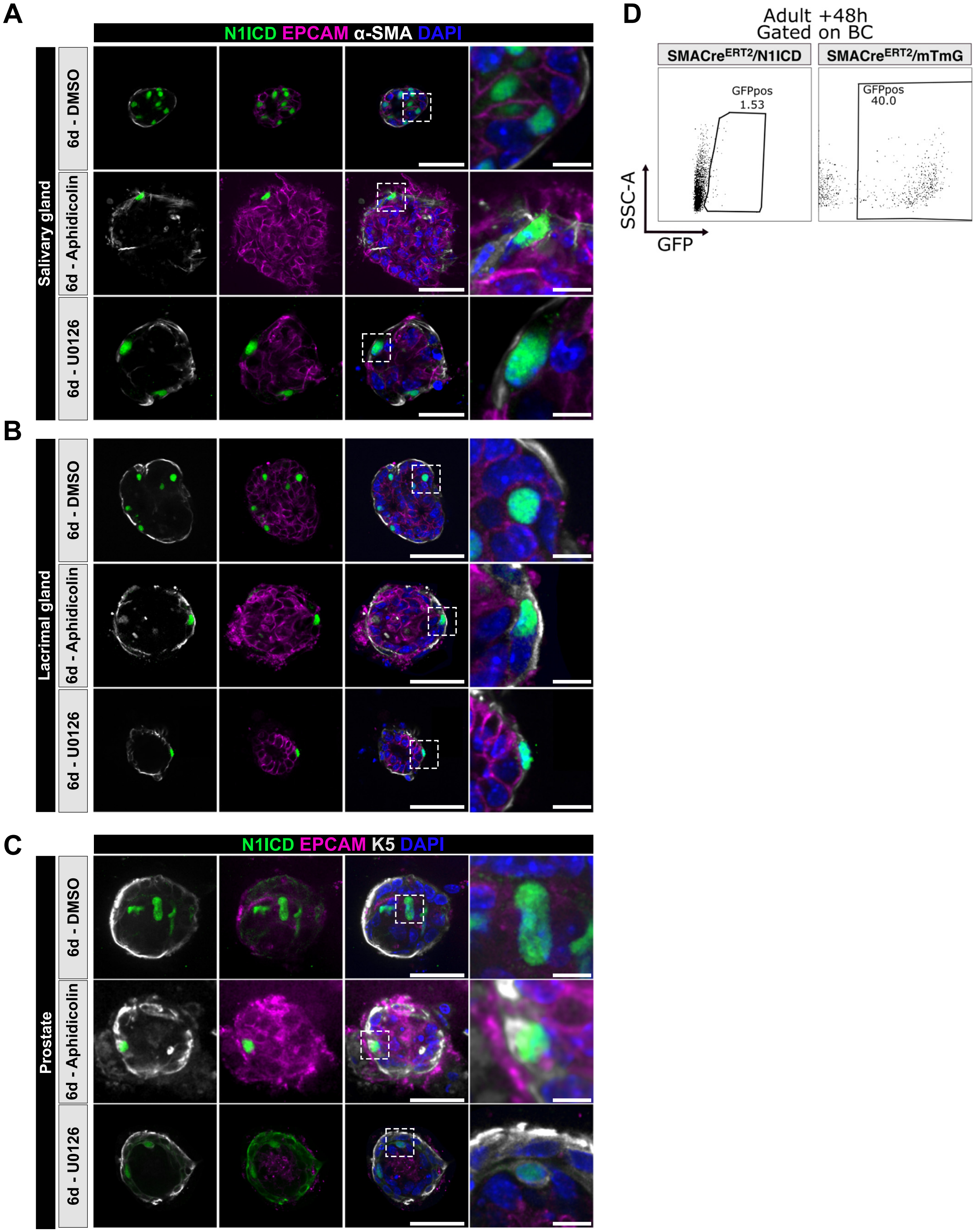
A
N1ICD EPCAM α-SMA DAPI
Salivary gland
6d - DMSO
6d - Aphidicolin
6d - U0126
B
Lacrimal gland
6d - DMSO
6d - Aphidicolin
6d - U0126
C
N1ICD EPCAM K5 DAPI
Prostate
6d - DMSO
6d - Aphidicolin
6d - U0126
D
Adult +48h
Gated on BC
SMACreERT2/N1ICD
SMACreERT2/mTmG
GFPpos
1.53
GFPpos
40.0
SSC-A
GFP

 © The Author(s)

Figure EV6. Proliferation is an obligatory step for lineage transition in salivary, lacrimal, and prostate organoids.

Related to Fig. 4. (**A–C**) Representative images showing immunofluorescence for nGFP (N1ICD in green), the luminal marker Epcam (purple) and the basal gene α-SMA (white) in SMACre^{ERT2}/N1ICD salivary (**A**) and lacrimal (**B**) gland organoids, or the basal gene K5 (white) in K5Cre^{ERT2}/N1ICD prostate (**C**) organoids treated with DMSO, Aphidicolin or U0126 for 6 days. Nuclei are stained with DAPI in blue. The scale bar represents 50 µm in (A–C) and 10 µm in the magnified insets. (**D**) Quantification of the proportion of basal GFP^{pos} cells after 48 h of induction in SMACre^{ERT2}/N1ICD and SMACre^{ERT2}/mTmG adult mice, showing the difference in recombination efficiency with the two Cre drivers.

© The Author(s)
